# Marine Biomass-Supported Nano Zero-Valent Iron for Cr(VI) Removal: A Response Surface Methodology Study

**DOI:** 10.3390/nano12111846

**Published:** 2022-05-27

**Authors:** Zhuang Tong, Qin Deng, Shengxu Luo, Jinying Li, Yong Liu

**Affiliations:** School of Science, Hainan University, Haikou 570228, China; 21074204@wit.edu.cn (Z.T.); 100002271@gxust.edu.cn (Q.D.)

**Keywords:** seashell resource utilization, pyrolysis, zero-valent iron, response surface methodology, chromium removal

## Abstract

Heavy metal ions such as Cr(VI) pose great hazards to the environment, which requests materials and methods for decontamination. Nano zero-valent iron (nZVI) has emerged as a promising candidate for Cr(VI) removal. Herein, harnessing the merits of marine biomass, a heterogeneous water treatment system for the decontamination of Cr(VI) is developed based on the in situ immobilization of nZVI on the seashell powder (SP)-derived porous support. A response surface methodology (RSM) study involving three independent factors is designed and conducted to direct material synthesis and reaction design for products with optimal performances. Under optimal synthetic conditions, the nZVI-loaded seashell powder (SP@nZVI), which is characterized in detail by scanning electron microscope (SEM), X-ray diffraction (XRD), and Fourier-transform infrared spectroscopy (FTIR), results in a 79% increase in the removal efficiency of Cr(VI) compared to free nZVI. Mechanism studies show that the removal of Cr(VI) by SP@nZVI conforms to the Langmuir adsorption model with a quasi-second order kinetic equation, in which redox reactions between nZVI and Cr(VI) occurred at the SP surface. The results of this work are expected to benefit the reuse of bioresource waste in developing environmental remediation materials.

## 1. Introduction

There have been frequent incidents of water pollution caused by harmful heavy metal ions around the world. In China, nearly 20% of water resources are contaminated by heavy metals such as chromium [1]. Cr(VI) is highly toxic and easily transferrable to humans through the food chain, which poses serious health risks to the environment. The treatment of Cr(VI) in the water is urgently required. Materials and methods for efficient Cr(VI) decontamination are highly requested [2]. Currently, methods for removing Cr(VI) from polluted water include ion exchange, membrane separation, chemical reduction, adsorption, and so forth [3]. Among them, adsorption is widely applied due to its easy operation and low cost [4]. However, the removal efficiency of surface adsorption still remains to be elevated. Besides, adsorption fails to convert Cr(VI) into its precipitable form, which is easier to be separated than the ionic form. Therefore, combining adsorption and other strategies may render improved performance of Cr(VI) decontamination.

Recently, nano zero-valent iron (nZVI), with strong REDOX activity and large specific areas, has shown great promise in removing heavy metal ions through a coupled adsorption–reduction mechanism [5]. However, conventionally designed nano zero-valent iron (nZVI) has two major drawbacks that may impair its applications [6]. (a) NaBH_4_, the commonly used reducing agent, is hazardous to both the human body and the environment [7]; (b) nZVI tends to form large aggregates in the reaction media due to strong particle–particle interactions. Therefore, the green synthesis of well-dispersed nZVI has long been under exploration. Compared to free nZVI, substrate-supported nZVI (SSnZVI) has been shown to possess improved stability, dispersibility, and reactivity during environmental remediation [8]. To make the synthesis of SSnZVI totally green and sustainable, the raw materials of both substrate materials and nZVI should be easily assessable, cost-effective, and safe to be processed. To this end, coupling the reutilization of bioresource waste with the production of functional SSnZVI is seemingly attractive.

China’s seafood production reached 64.453 million tons in 2019, of which shellfish production was around 15.28 million tons [9]. Consequently, a large quantity of seashell waste is produced, causing severe damage to the environment. Seashells mainly comprise calcium carbonate, though a small amount of organic matter also exists in them [10]. After pyrolysis at high temperatures, the organic matter in the seashell will disappear, leaving a porous tubular structure with greatly increased specific surface area [11]. Furthermore, the seashell surface possesses abundant negatively charged groups and can strongly bind cationic metal ions in solution [12]. All these properties of seashells make them excellent candidates for loading nZVI, rendering win–win cooperation between bioresource utilization and advanced material development, which, however, has not been explored.

Another problem concerning the development of SSnZVI lies in the huge amount of pre-experiments required for optimizing the synthetic conditions. To our knowledge, current studies on SSnZVI-enabled environmental applications rely on laborious optimization steps to approach the optimal material design. Response surface methodology (RSM) can dramatically reduce the workload of the optimization process by analyzing the relationships between response variables and factor variables based on a relatively small dataset [13]. It thus offers a promising tool to understand and even predict the potential impact of various factors on the performances of SSnZVI. 

**Figure 1:**
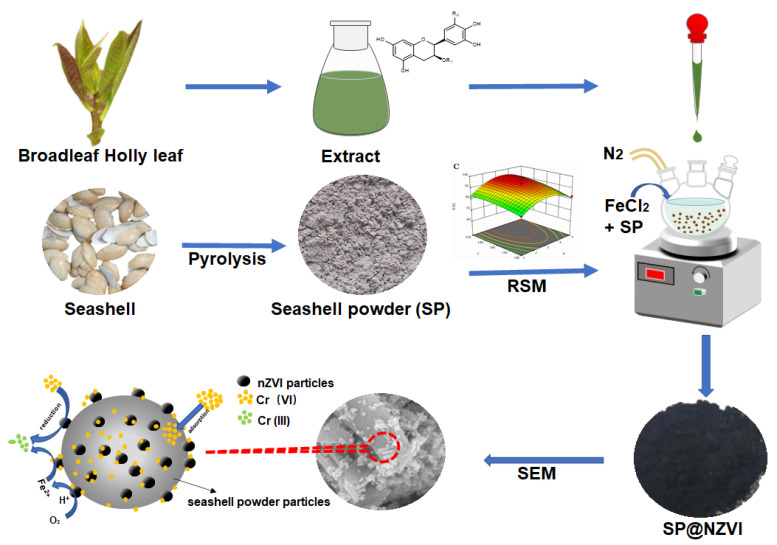
Proposed scheme for green synthesis of SP@nZVI and the related mechanism controlling its reaction with Cr(VI).

Inspired by the thought of “turning waste into wealth” and to make the material design process predictable, we propose an RSM-driven design of heterogeneous systems in which nZVI is immobilized in situ onto a porous support by a green wet chemical method. We select two bioresource wastes, the Broadleaf Holly leaf and the seashell, as the raw materials for preparing the reducing agent and the substrate, respectively. That is, the extract of Broadleaf Holly leaf is used to reduce ferrous ion into nZVI, and the pyrolyzed seashell powder (SP) serves as the substrate for immobilizing nZVI, yielding the SP@nZVI composite. Under the guidance of RSM, we monitor the potential impact of three independent factors (the mass ratio of SP/nZVI (A), the pyrolysis temperature (B), and the powder mesh of SP (C)) on the performances of SP@nZVI in removing Cr(VI) from water, which helps find the optimal design for detailed studies on the decontamination process. The design considerations of our work are summarized in Figure 1. Our results highlight the green concept of combining bioresource waste utilization with advanced materials development. Additionally, we demonstrate a de novo design strategy toward rapid optimization of the synthesis of SSnZVI for potential applications in environmental pollution control.

## 2. Materials and Methods

### 2.1. Chemicals and Materials

Broadleaf holly leaves were collected from a tea farm (Wanchang tea plantation, Haikou, China). Seashells were obtained from Yongxing Seafood Market (Haikou, China). Ferrous chloride tetrahydrate (FeCl_2_·4H_2_O) and diphenylamine urea were from Aladdin Industrial Corporation (Shanghai, China). Potassium dichromate[K_2_Cr_2_O_7_] and anhydrous ethanol were provided by Guangzhou Chemical Reagent Factory (Guangzhou, China). Phosphoric acid, sulfuric acid, and hydrochloric acid were supplied by Xilong Science (Guangzhou, China). Acetone was purchased from Yantai Yuandong Fine Chemical Co., Ltd. (Yantai, China). The above chemical reagents are all analytically pure.

### 2.2. Preparation of SP@nZVI

Fresh broadleaf holly leaves were washed with tap water to remove dust, then with distilled water and dried in an oven at 50 °C. Then, the leaves were cut into small pieces and sieved using a 2.5-millimeter sieve. In the Erlenmeyer flask, 10 g of leaves were added to 100 mL of distilled water, the solution was boiled at 65 °C for 90 min, followed by centrifuging at 8000 r min^−1^ for 10 min, and the supernatant was stored at 4 °C until used as a capping and reducing agent.

The seashells were collected from the seafood market and washed with tap water to remove dust; then, the seashells were immersed in 0.01 mol L^−1^ NaOH solution for 1 h to remove the stains on the seashell surface, washed with deionized water, and dried in a constant temperature oven. Finally, the seashells were placed in a muffle furnace and calcined at 300 °C, 500 °C, and 800 °C for 1 h, taken out for cooling, crushed, and sieved by sieve divided into 100 mesh, 160 mesh, and 220 mesh.

Fe(II) mixed solution was prepared by dissolving solid FeCl_2_·4H_2_O (5.49 g) in 100 mL of distilled water. SP with different parameters and Fe(II) mixed solution (100 mL) were placed into a three-necked open flask and stirred for 30 min. Subsequently, the extracting solution was added dropwise into the mixture, constantly stirring for 30 min. Finally, the black solid (SP@nZVI) was isolated by suction filtration and dried. The whole synthetic process was performed in the nitrogen atmosphere.

### 2.3. Characterization

The microstructures of the SP and SP@nZVI were imaged on G6 SEM (Pheno, Eindhoven, The Netherlands). FTIR spectra were obtained on a Tensor27 FTIR spectrometer (Bruker, Billerica, MA, USA) from 400 cm^−1^ to 4000 cm^−1^. XRD was recorded on a D8 Advance powder diffractometer (D8-ADVANCE, Bruker, Tokyo, Japan), operating at 40 kV and 40 mA.

### 2.4. Batch Experiment of Cr(VI) Removal

Batch experiments were conducted in a 150-milliliter conical flask with 50 mL of hexavalent chromium solution with a concentration of 10 mg L^−1^, and SP@nZVI was prepared under different conditions. The mixtures were placed on a rotary shaker at different temperatures. The residual concentrations of Cr(VI) were determined by a visible spectrophotometer at 540 nm according to the national standard at different time intervals such as 5, 10, 30, 50, 70, 90, 120, and 150 min. The concentration of Cr(VI) was analyzed by a UV Spectrophotometer (UV-2450, SHIMADZU, Kyoto, Japan) at a wavelength of 540 nm.

To evaluate the removal effect of SP@nZVI composite on Cr(VI) in water, the adsorption capacity or removal rate of the adsorbent is usually used. Thus, the study used the removal efficiency of Cr(VI) to describe the adsorption performance of SP@nZVI composites. The removal efficiency (R) of Cr(VI) adsorbed on SP@nZVI was calculated using Equation (1)

(1) $$R\left(\%\right)=\frac{C_{0}-C_{t}}{C_{0}}\times 100\%$$

where R is the removal efficiency of Cr(VI), %; C_0_ and C_t_ represent the initial concentration of the Cr(VI) solution and the residual concentration of Cr(VI), respectively, mg L^−1^.

The adsorption amount of Cr(VI) in an aqueous solution by the SP@nZVI is calculated using Equation (2)

(2) $$q=\frac{\left(C_{0}-C\right)\times V}{M}$$

where q (mg g^−1^) is the amount of adsorption, C_0_ (mg L^−1^) is the initial concentration of Cr(VI) before adsorption, and C (mg L^−1^) is the concentration of Cr(VI) after adsorption, V (L) is the volume of the solution, while M (g) is the mass of the SP@nZVI [14].

### 2.5. Experimental Design of Response Surface Methodology

To prepare SP@nZVI composite material with better performance to improve the removal efficiency of Cr(VI) and assess the effect of operational parameters on the response surface performance (Cr(VI) removal efficiency). In this study, the software Design-Expert 12.0 (State-Ease Inc., Minneapolis, MN, USA) was used to design the experiments and optimize the operating variables for Cr(VI) removal based on response surface methodology (RSM) [15]. Three main independent parameters such as the supported ratio of seashell powder to nano zerovalent iron (A), the pyrolysis temperature of shell powder (B), and the particle size of shell powder (C) are important factors for the preparation of SP@nZVI were examined at minimum (−1), medium (0), and maximum (+1) levels, plus five central points for determining the percentage of the sum of squares error. The range of parameters and levels used are presented in Table 1.

The second-order polynomial equation represents the relationship among dependent and independent variables (*β* is the response variable (Cr(VI) removal efficiency), which was calculated using Equation (3).

(3) $$\beta \left(\%\right)=\alpha_{0}+\overset{n}{\underset{i=1}{\sum }}\alpha_{i}X_{i}+\overset{n}{\underset{i=1}{\sum }}\alpha_{\mathrm{ii}}X_{ii}^{2}+\overset{n}{\underset{i=1}{\sum }}X_{i}\overset{n}{\underset{j=1}{\sum }}\alpha_{\mathrm{ii}}X_{i}+\varepsilon$$

where α is the coefficient; *X* was the independent variable, and ε was the random error [16].

Additionally, the validation of the proposed model was tested using analysis of variance (ANOVA), and the suitability of the model was evaluated by the values of R^2^, adequate precision, coefficient of variation, and lack of fit [17]. Additionally, the confirmatory experiments were conducted to validate predicted optimization values for Cr(VI) removal.

## 3. Results and Discussion

### 3.1. Synthesis and Characterization of SP@nZVI

**Figure 2:**
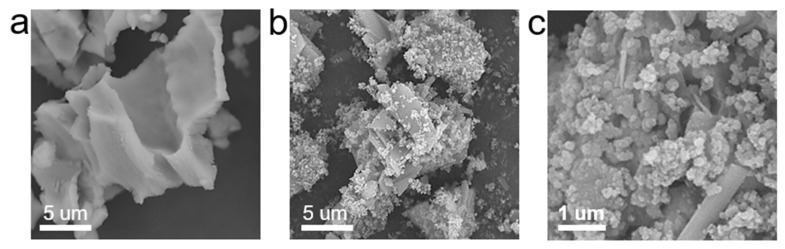
SEM images of SP (**a**), SP@nZVI particles (**b**) and SP@nZVI particles (**c**) at magnifications ×10,000, ×10,000, ×30,000 respectively.

A scanning electron microscope (SEM) showed that the pyrolyzed SP appeared as micrometer-scale fragments with porous surface structures (Figure 2a). After mixing with Fe(II) solution, the leaf extract was added to initiate the redox reaction. Since SP was porous and can efficiently bind Fe(II) through strong electrostatic interactions, abundant nZVI was observed on the surface of SP after the reaction, creating the SP@nZVI composite (Figure 2b). The SEM image at higher magnifications revealed that the generated nZVI had a well-defined spherical shape (Figure 2c). Meanwhile, the nZVI particles formed microscale clusters that tightly adhered to the SP surface; each cluster comprised tens of nZVI particles, rendering evenly distributed nZVI on the SP support. That is, SP can significantly reduce the particle agglomeration of nZVI by remodeling the particles into evenly distributed clusters.

**Figure 3:**
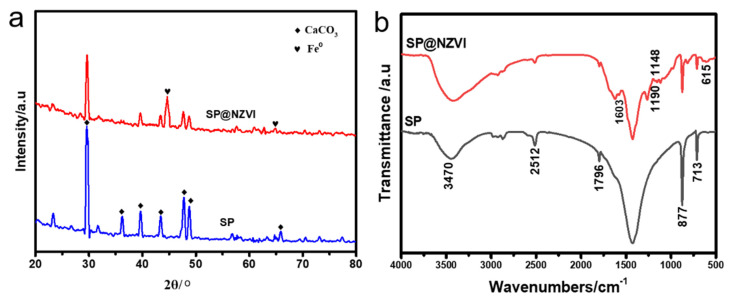
Characterization of SP and SP@nZVI: (**a**) XRD (**b**) FT−IR.

The XRD patterns of the pristine SP and SP@nZVI were shown in Figure 3a. SP had strong diffraction peaks at 2θ = 23.05°, 29.40°, 35.97°, 39.41°, 43.16°, 47.50°, and 48.07°. Based on Jade 6 software (Jade 6.0, 2010, MDI Materials Data, Livermore, CA, USA), these diffraction peaks were assigned to the different crystal planes of calcite-type calcium carbonate, indicating that the main component of SP is calcite-type calcium carbonate [18]. Compared with SP, SP@nZVI inherited the original diffraction peaks of SP, but the corresponding intensities were greatly weakened. This phenomenon can be explained by the shielding effect of the deposited nZVI on the SP surface. Furthermore, new diffraction peaks appeared in SP@nZVI at 44.67° and 65.02°, which were assigned to nZVI at (110) and (200) planes, respectively [19]. Interestingly, compared with nZVI synthesized by NaBH_4_, which typically showed diffraction peaks of iron oxides, our SP@nZVI had no such signals, suggesting the greatly reduced level of surface oxidation of nZVI. We hypothesized that the nZVI synthesized by leaf extract reduction was coated with a polyphenol-derived layer on its surface, which can alleviate the oxidation of the Fe(0) core [20].

Figure 3b showed the Fourier transform infrared (FTIR) absorption spectra of SP and SP@nZVI. We observed four characteristic peaks of carbonate ion in the SP sample at 2512, 1796, 877, and 713 cm^−1^ [21]. The peaks at 1796 and 1474 cm^−1^ were assigned to the anti-symmetric stretching vibration of the C−O bond [22]. The peaks at 877 cm^−1^ and 713 cm^−1^ peaks indicated the out-of-plane bending vibration and the in-plane bending vibration of the O−C−O bond, respectively. These results again supported the XRD results that SP was mainly composed of calcium carbonate [23]. Moreover, the peak at 3470 cm^−1^ corresponded to the stretching vibration of O−H and N-H bonds, which was attributed to the trace organic matters in SP [24,25]. Compared with SP, SP@nZVI also had the characteristic peaks of the carbonate ion, yet some new absorption features appeared in its FTIR spectrum. First, the characteristic peak at 3479 cm^−1^ broadened with stronger intensity, suggesting an increased amount of O−H and N−H bonds in SP@nZVI. The broadening of the absorption peak may be partially attributed to the association of O−H and N−H bonds [26]. Additionally, four new peaks appeared in the spectrum of SP@nZVI at 1603, 1190, 1148, and 615 cm^−1^. The two peaks at 1190 and 1148 cm^−1^ were from the stretching vibration of carboxyl groups, and the peak at 1603 cm^−1^ was from the stretching vibration of the benzene ring skeleton [27]. The peak at 615 cm^−1^ was considered to be the fingerprint of Broadleaf Holly leaf extract [28]. Taken together, we assumed that SP@nZVI formed an organic coating layer during the green reduction step mediated by the leaf extract.

### 3.2. Analysis of Variance and Validation of the RSM Model

We selected the efficiency of Cr(VI) removal (*β*) as the response variable and the mass ratio of SP/nZVI (A), the pyrolysis temperature (B), and the powder mesh of SP (C) as three independent factor variables. Note that for SP, a large value of powder mesh corresponded to a small powder size. Based on the Box–Behnken design (BBD), 17 test points, including 12 factorial experiments and 5 center tests, were traversed, and the dataset was shown in Table 2. The polynomial equation that described the relationship between *β* and factor variables A, B, and C was obtained using Design Expert 12 and expressed as follows: *β* = +93.34 + 2.38A − 3.36B + 3.26C − 1.17AB + 1.20AC + 0.66BC − 8.36A^2^ − 11.34B^2^ − 1.88C^2^.

**Figure 4:**
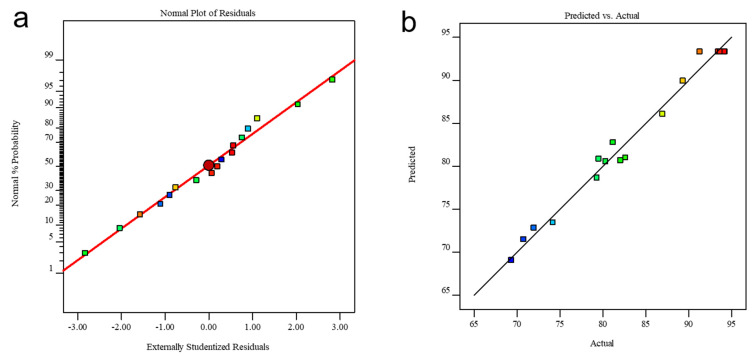
(**a**) The normal plot of residuals for Cr(VI) removal. (**b**) Plot of the actual and predicted response. The colors of points have no specific meanings.

The experimental data were statistically analyzed by analysis of variance (ANOVA) and the results were shown in Table 3. The regression model F-value of 49.36 and *p*-value < 0.0001 indicated that the quadratic model was statistically significant [29]. The *p*-value of the mismatch term was 0.1655 (*p* > 0.05), meaning that the unreasonable data was not significant, and the regression equation fitted well on the dataset. The *p*-value of single factors A, B, and C all appeared <0.05. Thus, these three factors had a significant impact on the removal efficiency of Cr(VI) [30]. Based on the F-value, we concluded that the influence level of the three factors followed the order B > C > A. That is, the pyrolysis temperature showed the biggest impact on Cr(VI) removal by SP@nZVI. Besides, the signal-to-noise ratio of the model was 19.651 (signal-to-noise ratio >4 is considered reasonable) and the coefficient of variation (C.V.) of the model was 1.94% (C.V. < 20% is considered credible). Thus, our model was statistically reasonable with high resolution and reliability. Collectively, the model was suitable for simulating the influence of the three synthetic conditions of SP@nZVI on the performance of Cr(VI) removal. To further evaluate the applicability of the quadratic model, we tested two types of diagnostic plots. Figure 4a showed the plot of normal probability versus residues. The data points significantly lay close to a straight line, indicating perfect normal distributions of residues as well as the accuracy of the assumptions. Figure 4b showed the relationship between the predicted and actual values of the efficiency of Cr(VI) removal. The predicted values were close to the actual values for each test point on a straight line, indicating the statistical validation of the model. Collectively, ANOVA validated the applicability of our model in simulating the collective impact of the three factors on Cr(VI) removal [31].

### 3.3. Interaction Effects of Variable Factors

**Figure 5:**
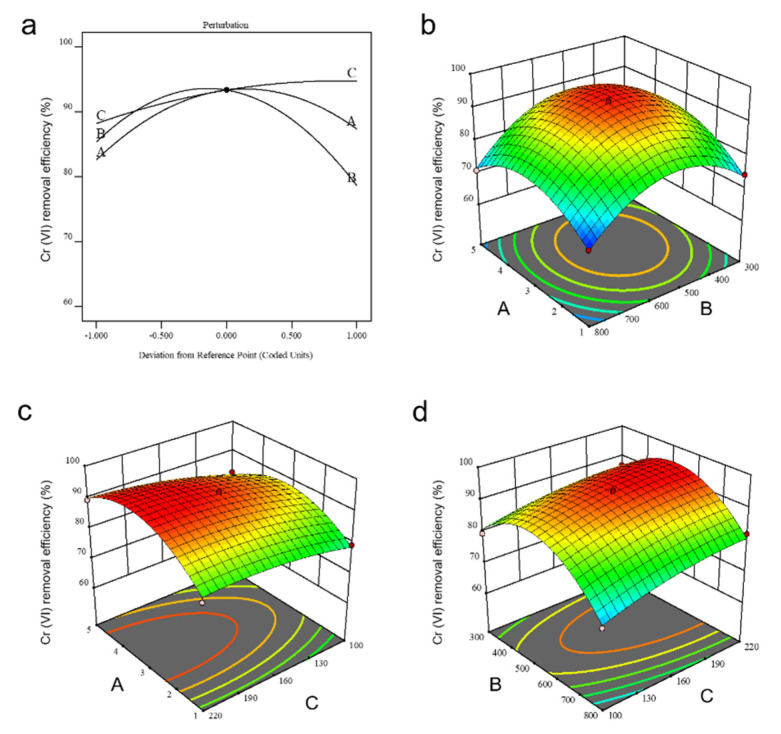
Surface for the effect of different variables on Cr(VI) removal. (**a**) The independent impact of three variables A, B, and C on Cr(VI) removal. A, B, and C indicate the mass ratio of SP/nZVI, the pyrolysis temperature, and the powder mesh of SP, respectively. (**b**–**d**) The impact of combinations of every two of the three variables on Cr(VI) removal: A and B (**b**), A and C (**c**), B and C (**d**).

We next studied the interaction effect between any two of the three independent factors A, B, and C, as well as the impact of such interactions on the response factor *β*. First, Figure 5a presented the impact of varying a single synthetic condition on the efficiency of Cr(VI) removal. With the increase of pyrolysis temperature (B) and the mass ratio of SP/nZVI (A), the efficiency of Cr(VI) removal by the obtained SP@nZVI increased first and then decreased, showing a maximum value at the center point. However, the powder mesh of SP (C) had a relatively small effect on the performance of SP@nZVI, and the larger the powder mesh (corresponding to smaller particle size), the higher the efficiency of Cr(VI) removal. Figure 5b–d were the three-dimensional response surface diagrams showing the collective impact of A–B, A–C, and B–C on the efficiency of Cr(VI) removal. From these pictures, we summarized several rules that contributed to the optimal performance of Cr(VI) removal. As shown in Figure 5b, the contour plot involving factors A–B resembled a set of concentric circles. Therefore, if one factor was fixed, there should be a maximum value *β*_max_ near the central point of the variation range of the other factor, which was in good consistent with the parabolic response curves of A and B in Figure 5a. For example, at a certain pyrolysis temperature (B), the increased efficiency of Cr(VI) removal accompanying the increase of the mass ratio of SP/nZVI (A) from 1:1 to 3:1 was explained by the increasingly large surface area provided by the SP, which can benefit the uniform dispersion of nZVI as well as the adsorption of Cr(VI) in the solution. However, as the mass ratio of SP/nZVI (A) reached over 3:1, the concentrated SP may aggregate into large sediments and the surface area for nZVI loading and Cr(VI) adsorption thereby decreased. Similarly, at a preset mass ratio of SP/nZVI (A), as the pyrolysis temperature (B) increased from 300 to 500 °C, the organic matter in the SP gradually decomposed upon heating, exposing increasing areas of the original porous surface of SP. Thus, the efficiency of Cr(VI) removal gradually increased in this range of pyrolysis temperature. As the pyrolysis temperature (B) further increased to over 600 °C, the efficiency of Cr(VI) removal decreased because, at such high temperatures, the CaCO_3_ matrix will be thermally decomposed into CaO and the SP will lose its original tubular structure for the efficient loading and dispersion of nZVI. This role, namely the parabola rule, also works for factors A and B (but not for C) in couples A–C and B–C, as shown in Figure 5c,d, respectively. In Figure 5c,d, the contour plot appeared as a set of semicircles that were symmetrical along the C axis. Thus, the powder mesh of SP (C) had a monotonic impact on the response variable *β* within the test range (also see plot C in Figure 5a). Specifically, at either a preset value of the mass ratio of SP/nZVI (A) or the pyrolysis temperature (B), increasing the powder mesh of SP (C) resulted in increased efficiency of Cr(VI) removal. Since the larger powder mesh corresponded to the smaller powder size, the improved Cr(VI) removal can be explained by the enhanced specific surface area of SP with smaller sizes, which favored the uniform dispersion of nZVI and the efficient interactions with Cr(VI).

Based on the above analysis, Design Expert 12 theoretically gave the optimal synthetic condition: the mass ratio of SP/nZVI (A) 3.43, the pyrolysis temperature of 516.79 °C, and the size of SP 210 mesh, yielding a maximum removal efficiency of 95.309%. Therefore, in the following experiments, to study the details of SP@nZVI-mediated removal of Cr(VI), we set the values of A, B, and C to be 3.43, 517 °C, and 210 mesh, respectively. We performed three parallel experiments under this condition, and the average value of Cr(VI) removal efficiency was 97.032%, which was slightly higher than the predicted value. In short, the experimental optimization by BBD has been proved effective in guiding the design of SP@nZVI for optimal performances in Cr(VI) removal.

### 3.4. Cr(VI) Removal Performance

#### 3.4.1. Efficiency of Cr(VI) Removal

**Figure 6:**
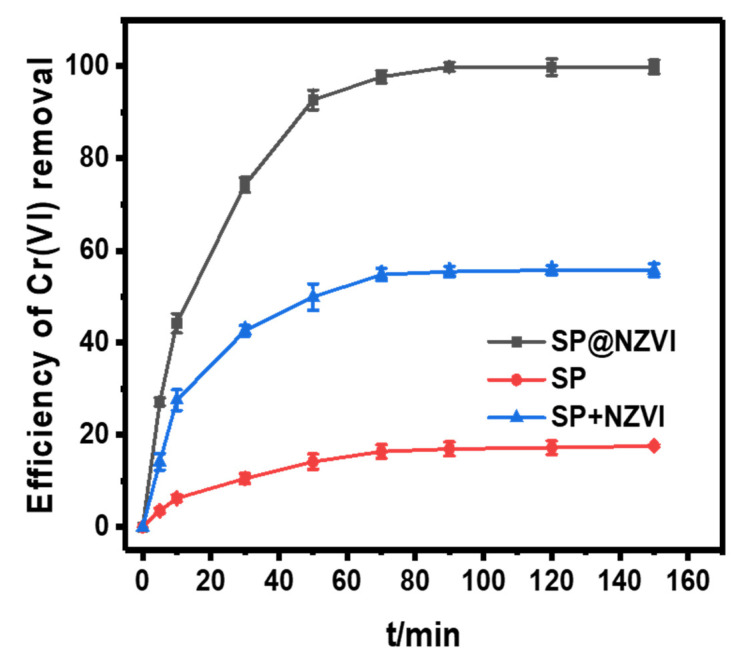
The effect of different materials on Cr(VI) removal.

We evaluated the performance of SP@nZVI prepared under the optimal synthetic conditions in removing Cr(VI) from water: at T = 25 °C and pH = 5.0, 100 mL Cr(VI) solutions (30 mg L^−1^) were treated with SP, SP + nZVI (physically mixed SP and nZVI with identical mass ratio of SP/nZVI to SP@nZVI), and SP@nZVI, and the efficiency of Cr(VI) removal was continuously monitored in 150 min (Figure 6). For the three tested materials, the efficiency of Cr(VI) removal at equilibrium was 17.95% (SP), 55.75% (SP + nZVI), and 99.85% (SP@nZVI), respectively, suggesting the synergistic enhancement of Cr(VI) removal capacity in the SP@nZVI composite. At pH 2–6, both Cr_2_O_7_^2−^ and HCrO_4_^−^ existed in the solution, and CrO_4_^2−^ became dominant when pH > 6. The higher Cr(VI) removal efficiency of SP@nZVI than that of SP + nZVI was possibly due to (i) the increased dispersity of nZVI on the SP support, and (ii) the shortened path of mass transfer at the SP/nZVI interface after they formed composites. The maximum capacity of Cr(VI) adsorption on SP and SP@nZVI was determined to be 5.39 mg g^−1^ and 29.96 mg g^−1^, respectively, which appeared higher than reported values using biochar and magnetic biochar adsorbents (Table 4). 

#### 3.4.2. Adsorption Isotherms

**Figure 7:**
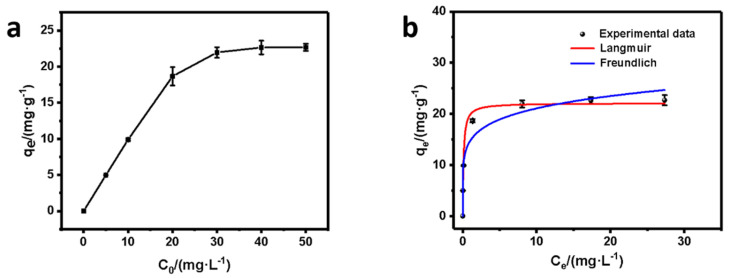
Adsorption isotherms of SP@nZVI. (**a**) Variation of the equilibrium uptake amount with increased initial Cr(VI) concentration. (**b**) Non−linear fitting using Langmuir and Freundlich adsorption models.

To further investigate the Cr(VI) removal process, we monitored the adsorption isotherms of SP@nZVI by varying the initial concentration of Cr(VI). Figure 7a depicted the relationship between the initial concentration of Cr(VI) (C_0_, mg L^−1^) and the adsorption amount of Cr(VI) at equilibrium by SP@nZVI (*q_e_*, mg g^−1^). The results showed that *q_e_* linearly increased as C_0_ increased from 5 to 30 mg L^−1^. However, as C_0_ was higher than 30 mg L^−1^, *q_e_* gradually reached an equilibrium maximum at 23.66 mg g^−1^, indicating that the active sites on the SP@nZVI composite tended to be saturated by C_0_ larger than 30 mg L^−1^. Based on Figure 7a, we fitted the experimental data with both Langmuir and Freundlich adsorption isotherms. The Langmuir model is presented as Equation (4).

(4) $$q_{e}=\frac{K_{L}q_{\max}C_{e}}{1+K_{L}C_{e}}$$

where *q_e_* (mg g^−1^), *q_max_* (mg g^−1^), *C_e_* (mg L^−1^), and *K_L_* (L mg^−1^) were the uptake amount of Cr(VI) at equilibrium, Langmuir maximum adsorption capacity, Cr(VI) concentration at equilibrium, and Langmuir constant, respectively. 

The Freundlich model is presented as Equation (5).

(5) $$q_{e}=K_{F}C_{e}^{{1/n}_{F}}$$

where *K_F_* and n*_F_* were Freundlich constant and adsorption intensity, respectively.

The fitting results were shown in Figure 7b and Table 5. The correlation coefficients R^2^ were 0.9824 and 0.9412 for the Langmuir model and the Freundlich model respectively, indicating that the adsorption of Cr(VI) mainly occurred at the heterogeneous surface of SP@nZVI as a monolayer adsorption model [37]. Besides, the maximum adsorption capacity of SP@nZVI to Cr(VI) was estimated to be 22.11 mg·g^−1^ based on the Langmuir model.

#### 3.4.3. Adsorption Kinetics

**Figure 8:**
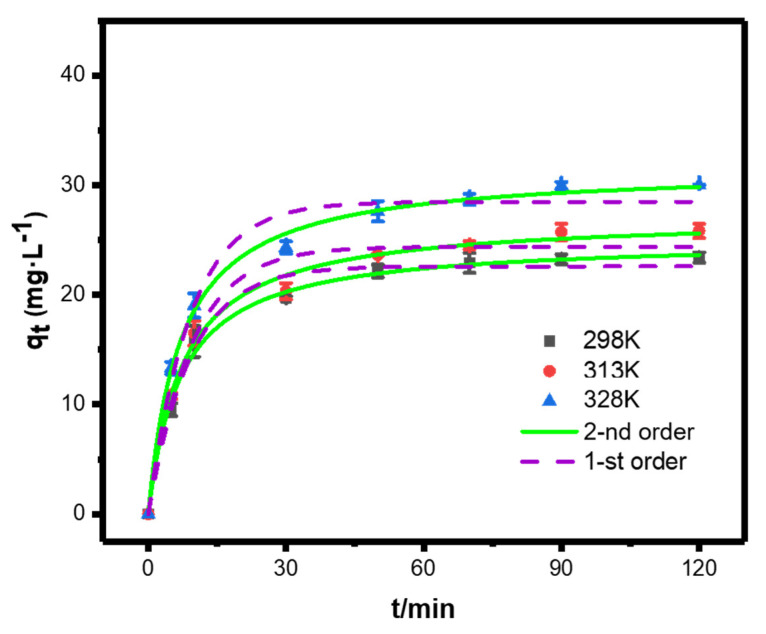
Pseudo first-order and pseudo second-order kinetic model fitting of the adsorption kinetics of SP@nZVI at different temperatures.

We next studied the adsorption kinetics of SP@nZVI-mediated Cr(VI) removal. As shown in Figure 8, the adsorption amount of Cr(VI) at equilibrium (*q_e_*, mg g^−1^) increased from 20.41 to 29.95 mg g^−1^ as the temperature increased from 298 to 328 K. The experimental results were examined using kinetic models of pseudo first-order (PFO) and pseudo second-order (PSO) to help understand the mechanisms of Cr(VI) removal. The non-linear forms of PSO and PFO models were presented as Equations (6) and (7), respectively.

(6) $$q_{t}=q_{max}\left(1-e^{-k_{1}t}\right)$$

(7) $$q_{t}=\frac{k_{2}q^{2}{}_{max}t}{1+k_{2}q_{max}t}$$

where *t* was the reaction time, *q_e_* (mg g^−1^) was the adsorption amount of Cr(VI) at equilibrium, *q_t_* (mg g^−1^) was the adsorption amount at time (*t*), *k*_1_ (min^−1^) and *k*_2_ (g mg^−1^ min^−1^) were the rate constants of PFO and PSO, respectively. The fitting curves as well as the kinetic parameters were presented in Figure 8 and Table 6. The results indicated that the adsorption of Cr(VI) by SP@nZVI followed a PSO model due to the larger R^2^. Thus, the removal of Cr(VI) was more likely controlled by chemisorption [38]. Collectively, SP@nZVI showed excellent performances in removing Cr(VI) from water, which was foremostly attributed to heterogeneous chemisorption at the solid–liquid interface.

### 3.5. Mechanism Study by XPS

**Figure 9:**
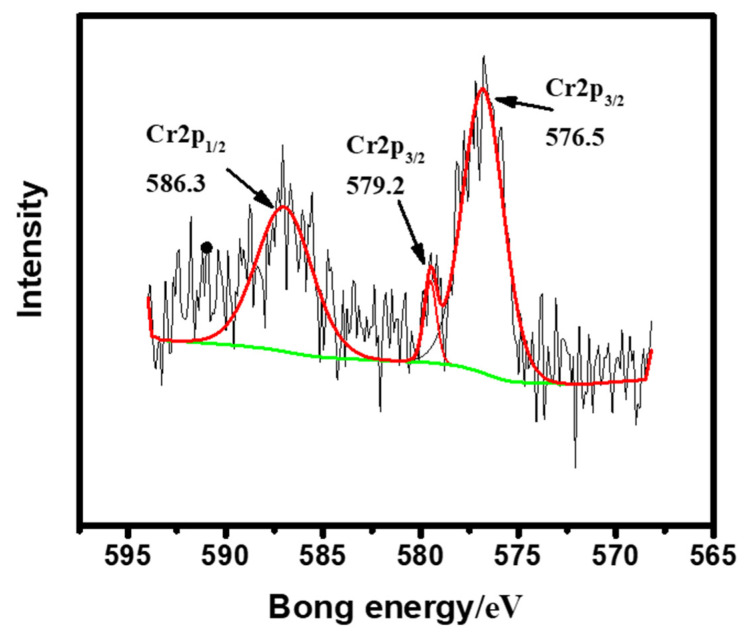
XPS patterns of SP@nZVI. The red and green lines are the fitted curve of raw data and the baseline, respectively.

To further illustrate the mechanism of Cr(VI) removal, we characterized the XPS of SP@nZVI before and after the adsorption experiments. The content of different elements (atomic ratio) in SP@nZVI was analyzed and shown in Table 7. After incubation with Cr(VI) solutions, the Fe content in SP@nZVI decreased from 20.14% to 17.54%, yet the Cr content increased from 0 to 1.63%. This result strongly indicated that during reactions, some Fe in nZVI dissolved and escaped into the bulk solution, and meanwhile, some Cr was “immobilized” onto the surface of SP@nZVI. Figure 9 presented the Cr 2p spectrum, in which three characteristic peaks were observable at 586.3, 579.2, and 576.5 eV. Specifically, the peak at 586.3 eV was assigned to Cr(VI), and the peaks at 579.2 and 576.5 eV corresponded to Cr(III) [39]. Besides, the contributions of different Cr species to the Cr 2p spectrum were 60.96% and 39.04% for Cr(III) and Cr(VI), respectively. Thus, the Cr species enriched by SP@nZVI mainly appeared as Cr (III), meaning that reduction processes occurred during Cr(VI) removal by SP@nZVI. Based on these results, we presumed a multichannel mechanism for Cr(VI) removal that the decontamination process was enabled by (i) physical adsorption by the porous microstructures of SP@nZVI and (ii) subsequent reduction of Cr(VI) by Fe(0) into less soluble Cr(III) species [40,41].

## 4. Conclusions and Future Perspectives

To summarize, we have made successful attempts in the reutilization of seashell wastes by pyrolyzing them into porous support materials and applying them for nZVI-based Cr(VI) removal. The performance of SP@nZVI in Cr(VI) removal is demonstrated to be highly predictable using BBD-enabled RSM analysis, implying the great power of mathematical models in driving material design. The optimized design, in which SP@nZVI is synthesized with a mass ratio of SP/nZVI of 3.43, a pyrolysis temperature of 516.79 °C, and a SP size of 210 mesh, yields a maximum removal efficiency of 95.309%. 

Some issues in this work remain to be addressed in future studies. The detailed mechanisms of Cr(VI) decontamination are still elusive. Based on our results, at least two pathways, adsorption and reduction, are involved in Cr(VI) removal. However, the morphology and property of the immobilized Cr on SP@nZVI are not investigated. The details of electron transfer between nZVI and Cr remain unknown as well. Besides, since only three parameters are involved in our RSM dataset, the extended applications of RSM in complicated reaction systems with more variables (such as the types of iron salts and the size of nZVI particles) are highly anticipated.

## Figures and Tables

**Table 1:** Experimental ranges and levels of independent variables.

Independent Factors	Symbols	Levels		
		−1	0	+1
Supported ratio	A	1:1	3:1	5:1
Pyrolysis temperature (°C)	B	300	550	800
Seashell powder size (mesh)	C	100	160	220

**Table 2:** Box-Behnken experimental design and response values.

Number	A	B	C	Measure Value/(%)	Predictive Value/(%)
1	1:1	550	220	81.17	82.78
2	3:1	800	220	82.04	80.68
3	3:1	550	160	94.15	93.34
4	3:1	300	100	79.51	80.87
5	1:1	550	100	79.29	78.66
6	3:1	550	160	93.44	93.34
7	3:1	800	100	71.95	72.83
8	5:1	550	220	89.31	89.94
9	5:1	300	160	80.30	80.55
10	3:1	550	160	91.28	93.34
11	1:1	800	160	69.32	69.07
12	3:1	550	160	93.64	93.34
13	3:1	550	160	94.19	93.34
14	3:1	300	220	86.95	86.07
15	5:1	800	160	70.75	71.48
16	5:1	550	100	82.62	81.01
17	1:1	300	160	74.18	73.45

**Table 3:** Analysis of variance for the fitted quadratic model.

Source	Sum of Squares	Degrees of Freedom	Mean Square	F Value	*p* Value Prob > F	Significance
Model	1151.76	9	127.97	49.36	<0.0001	significant
A	45.22	1	45.22	17.44	0.0042	-
B	90.32	1	90.32	34.84	0.0006	-
C	85.15	1	85.15	32.84	0.0007	-
AB	5.50	1	5.50	2.12	0.1886	-
AC	5.78	1	5.78	2.23	0.1789	-
BC	1.76	1	1.76	0.68	0.4377	-
A^2^	294.18	1	294.18	113.47	<0.0001	-
B^2^	541.81	1	541.81	208.98	<0.0001	-
C^2^	14.94	1	14.94	5.76	0.0474	-
Residual	18.15	7	2.59	-	-	-
Lack of fit	12.43	3	4.14	2.90	0.1655	not significant
Pure error	5.72	4	1.43	-	-	-
Cor. Total	1169.91	16	-	-	-	-

Q-Squared: 0.9845, Adeq Precisior: 19.651, C.V. = 1.94%.

**Table 4:** Comparison of adsorption capacities of Cr(VI) ions on SP and SP@nZVI with reported adsorbents.

Adsorbent	Modified Method	*q*_*m*_ (mg·g^−1^)	Reference
MCCS	corn cob silica supported nanoscale zero-valent iron	11.1	[32]
nZVI/ATP	attapulgite (ATP) maintained nZVI	22.01	[33]
SS/nZVI	magnetic biochar prepared by co-pyrolysis of nZVI and sewage sludge	11.56	[34]
BTS	Biochar derived from tobacco stems	3.84	[35]
Fe@PC	PC modified with Fe(NO_3_)_3_	10.07	[36]
PC	Porous carbon	2.50	[36]
SP	High-temperature pyrolysis seashell powder	5.39	This study
SP@nZVI	Marine Biomass-Supported nZVI	29.96	This study

**Table 5:** Adsorption isotherm fitting constant.

Adsorption Isotherm Model	Model Parameter	R^2^
Langmuir	*q*_*m*_ = 22.11 ± 1.14; *K*_*L*_ = 8.61 ± 1.84	0.9824
Freundlich	n*_F_* = 6.39; *K*_*F*_ = 14.70 ± 1.14	0.9412

**Table 6:** Kinetic model fitting constants at different temperatures.

Temperature/K	PFO			PSO		
	*k*_1_/min^−1^	*q_e_*/mg·g^−1^	R^2^	*k*_2_/g·mg^−1^·min^−1^	*q_e_*/mg·g^−1^	R^2^
298	0.10976 ± 0.01082	22.595 ± 0.47221	0.98598	5.65 × 10^−3^ ± 5.996 × 10^−4^	25.044 ± 0.44297	0.99448
313	0.10651 ± 0.01519	24.392 ± 0.7398	0.97078	4.95 × 10^−3^ ± 5.866 × 10^−4^	27.1868 ± 0.54506	0.99308
328	0.11024 ± 0.0143	28.48417 ± 0.78278	0.97537	4.51 × 10^−3^ ± 3.778 × 10^−4^	31.5862 ± 0.4404	0.9965

**Table 7:** Element content of SP@nZVI before and after adsorption.

Sample	Elemental Contents (%)				
	C	O	Fe	Ca	Cr
before adsorption/%	22.45	55.52	20.14	1.88	0
after adsorption/%	28.96	50.16	17.54	1.71	1.63

## Data Availability

Not applicable.
